# Effect of Fiber Blending Ratio on the Tensile Properties of Steel Fiber Hybrid Reinforced Cementitious Composites under Different Strain Rates

**DOI:** 10.3390/ma14164504

**Published:** 2021-08-11

**Authors:** Minjae Son, Gyuyong Kim, Hongseop Kim, Sangkyu Lee, Yaechan Lee, Jeongsoo Nam, Koichi Kobayashi

**Affiliations:** 1 Department of Architectural Engineering, Chungnam National University, 99 Daehak-ro, Yuseong-gu, Daejeon 34134, Korea; minjae931226@naver.com (M.S.); lsg2357@naver.com (S.L.); cks6832@naver.com (Y.L.); j.nam@cnu.ac.kr (J.N.); 2 Building Safety Research Center, Department of Living and Built Environment Research, Korea Institute of Civil Engineering and Building Technology, 283 Goyang-daero, Ilsanseo-gu, Goyang-si 10223, Korea; hongseopkim@kict.re.kr; 3 Department of Civil Engineering, Faculty of Engineering, Gifu University, 1-1 Yanagido, Gifu 501-1193, Japan; ko2ba@gifu-u.ac.jp

**Keywords:** fiber blending ratio, strain rate, hooked steel fiber, smooth steel fiber, synergistic effect

## Abstract

In this study, a high-performance hybrid fiber-reinforced cementitious composite (HP-HFRCC) was prepared, by mixing hooked steel fiber (HSF) and smooth steel fiber (SSF) at different blending ratios, to evaluate the synergistic effect of the blending ratio between HSF and SSF and the strain rate on the tensile properties of HP-HFRCC. The experimental results showed that the micro- and macrocrack control capacities of HP-HFRCC varied depending on the blending ratio and strain rate, and the requirement for deriving the appropriate blending ratio was confirmed. Among the HP-HFRCC specimens, the specimen mixed with HSF 1.0 vol.% and SSF 1.0 vol.% (H1.0S1.0) exhibited a significant increase in the synergistic effect on the tensile properties at the high strain rate, as SSF controlled the microcracks and HSF controlled the macrocracks. Consequently, it exhibited the highest strain rate sensitivities of tensile strength, strain capacity, and peak toughness among the specimens evaluated in this study.

## 1. Introduction

High-performance fiber-reinforced cementitious composites (HPFRCCs) have attracted considerable attention because they significantly improve energy absorption capacities by exhibiting strain-hardening behavior, accompanied by multiple cracks, owing to the bridging effect of fibers under quasi-static tensile loads [1,2,3,4,5]. In particular, HPFRCC is mentioned as one of the materials that can improve the resistance of civil infrastructure at extreme loads (i.e., earthquakes, explosions, and impacts) [6]. Furthermore, according to recent research results, HPFRCC is a promising material with high potential to improve the fire resistance and seismic performance of structures [7,8,9,10]. As such, various studies have been conducted to effectively improve the performance of HPFRCC, and the representative performance improvement methods are as follows: improvement of the bonding strength between the fiber and matrix by increasing the matrix strength [11], improvement of the interfacial adhesive bonding between the fiber and matrix through the use of high-performance synthetic fibers [12], improvement of the performance of the matrix by adding nanomaterials [13], and improvement of the crack bridging performance of the matrix using steel fibers that are deformed into specific shapes [14]. 

Among them, the high-performance hybrid fiber-reinforced cementitious composite (HP-HFRCC), in which two or more types of fibers with different physical properties, lengths, and shapes are mixed, exhibits excellent performance even at a low fiber volume fraction depending on the combination of fibers; therefore, continuous research has been conducted on HP-HFRCC [15,16,17,18,19,20]. In particular, it was reported that the use of both long and short fibers exhibits high flexural and tensile strengths and energy absorption capacity because each fiber effectively controls microcracks and macrocracks even at a fiber volume fraction of 2.0 vol.% or less [21,22,23,24,25]. Recent research has reported that the tensile behavior of HPFRCC varies depending on the type of reinforcing fiber under high strain rates [26,27,28,29,30]. However, there is a need to further investigate whether the excellent flexural and tensile strengths and energy absorption capacity of HP-HFRCC under quasi-static load conditions are still maintained under high strain rate conditions. Therefore, studies on the tensile behavior of HP-HFRCC have been conducted based on the strain rate.

Tran et al. [31] investigated hybrid reinforcement with long and short fibers as a method to effectively improve the performance of ultra-high-performance fiber-reinforced concrete (UHPFRC) even at a low fiber volume fraction. They analyzed the fiber blending effect of the smooth steel fiber (SSF), which is a short fiber, on the tensile properties of ultra-high-performance hybrid fiber-reinforced concrete (UHP-HFRC) containing different types of long fibers at different strain rates. They reported that a cementitious composite mixed with a long SSF (length: 30 mm; diameter: 0.3 mm) and medium-length SSF (length: 19 mm and diameter: 0.2 mm) exhibited the highest synergistic effect on the strain capacity and peak toughness at a high strain rate, owing to an improvement in the bonding strength between the fiber and matrix. Park et al. [32] investigated the effect of matrix strength on the tensile behavior of the strain-hardening fiber-reinforced cementitious composite (SH-FRCC) that was hybrid-reinforced with steel fibers. They fabricated SH-FRCC specimens that were reinforced with hooked steel fibers (HSFs) as long fibers and SSFs as short fibers, with the matrix strengths of 56, 81, and 180 MPa, respectively, and analyzed them. As the strain rate increased, the number of non-straightened HSFs increased for low matrix strength owing to the microcracks generated in the matrix around the HSF. However, for high matrix strength, damage to the matrix was reduced and the number of non-straightened HSFs decreased. Based on the tensile behavior of SH-FRCC, it was confirmed that a high matrix strength is favorable for improving the tensile strength and energy absorption capacity; nonetheless, a low matrix strength is effective in terms of the strain capacity.

Tran et al. [33] evaluated the impact of the rate on the fracture energy of UHPFRC. They also evaluated the effect of fiber blending on the fracture energy of UHPFRC at high rates. UHP-HFRC, hybrid-reinforced with long and short fibers, did not exhibit significant tensile strength improvement compared with the ultra-high-performance mono fiber-reinforced concrete (UHP-MFRC) that was reinforced with medium-length steel fiber. UHP-HFRC, however, exhibited a significant improvement in strain capacity and energy absorption capacity compared with UHP-MFRC owing to the microcrack control effect of short fibers and the macrocrack control effect of long fibers. Finally, they concluded that UHP-HFRC was more favorable than UHP-MFRC for improving the dynamic increase factors (DIFs) of the peak toughness (UHP-MFRC: 1.2 to 2.8; UHP-HFRC: 4.1 to 4.6) and softening toughness (UHP-MFRC: 0.6 to 0.8; UHP-HFRC: 0.8 to 1.1). Maalej et al. [34] investigated an engineered cementitious composite (ECC) for its application to protective structures because it exhibits tensile strain-hardening and multiple cracking behavior. Dynamic tensile and impact tests of hybrid-fiber ECC were conducted to effectively improve the tensile strength and strain capacity. They reported that hybrid-fiber ECC exhibited multiple cracking behavior within all strain rate ranges (2 × 10^−6^ to 2 × 10^−1^/s); however, there was no significant change in the strain capacity, unlike the tensile strength, which improved as the strain rate increased.

Zhao et al. [18] expected that the impact resistance of an ultra-high toughness cementitious composite (UHTCC) could be improved through the hybridization of steel fibers with polyvinyl alcohol fiber (PVA). They investigated the splitting tensile behavior of hybrid-fiber UHTCC under dynamic tensile loading to identify the individual roles of hybrid fibers that contributed to the impact resistance of UHTCC. The results of the splitting tensile test of hybrid-fiber UHTCC showed that the tensile strength increased as the steel fiber content increased under quasi-static loads; however, the energy absorption capacity decreased as the steel fiber content increased from 0.5% to 1.5%. However, under dynamic tensile loads, the tensile strength and energy absorption capacity of the UHTCC significantly increased as the steel fiber content increased. They reported that the optimal hybrid fiber blending ratio could vary depending on the strain rate because the tensile strength and Young’s modulus of the fiber as well as the interfacial adhesive bonding between the fiber and matrix exhibited different strain rate sensitivities. Tran et al. (2021) [35] proposed mixing SSF with polyamide fiber (PA) to reduce the amount of steel fiber used, thereby securing the economic efficiency of UHP-HFRC. They investigated the tensile resistance of UHP-HFRC that used SSF and PA as long and short fibers, respectively, according to the strain rate. They found that mixing long SSF with short SSF was effective in improving the tensile strength; nonetheless, mixing long SSF with short PA was effective in improving the strain capacity and peak toughness. They also reported that using 1% long fiber and 1% short fiber slightly improved the tensile strength of UHP-HFRC compared with using 1% long fiber and 0.5% of short fiber; nonetheless, it decreased the strain capacity and peak toughness, and was particularly negative for the DIFs of tensile properties. 

The literature review shows that various studies have been conducted on the tensile behavior of HP-HFRCC based on the strain rate. Most studies have reported that the tensile behavior of HP-HFRCC varies depending on the strain rate. Nonetheless, most of the studies have been focused on evaluating the tensile behavior of HP-HFRCC based on the type of fiber mixed or the matrix strength. Furthermore, the studies on the blending ratio of fiber are insufficient. Because the tensile behavior of HP-HFRCC varies depending on the blending ratio of hybrid fibers [36], it is necessary to examine the fiber blending ratio that can effectively improve performance by evaluating the influence of the strain rate on the tensile behavior of HP-HFRCC with different fiber blending ratios [37,38]. 

Therefore, in this study, the effect of the blending ratio between long and short fibers on the tensile behavior of HP-HFRCC was evaluated under different strain rates. In particular, the synergistic effect of the blending ratio of long and short fibers on the tensile properties of HP-HFRCC was analyzed at a fixed fiber volume fraction (2.0%) to examine the optimal fiber blending ratio to realize an excellent performance. Accordingly, HP-HFRCC was fabricated using HSF as a long fiber and SSF as a short fiber and by adjusting the fiber blending ratio. Through the direct tensile test of HP-HFRCC under static-rate and high-rate conditions, the stress–strain curve, tensile strength, strain capacity, peak toughness, and softening toughness were evaluated, and the synergistic effect on tensile properties, according to the strain rate and fiber blending ratio, was analyzed. In addition, the multiple cracking behavior and fiber failure behavior were also evaluated to analyze the tensile behavior of HP-HFRCC.

## 2. Experimental Design and Method

### 2.1. Experimental Plan

In a previous study [37], the failure behavior of PVA was found to vary depending on the strain rate in HP-HFRCC mixed with HSF and PVA. Therefore, the microcrack control effect decreased as the volume fraction of PVA increased at the high strain rate. At the high strain rate and static rate, new short fibers are required for the effective control of microcracks in HP-HFRCC. According to previous studies [31,32,33], the use of SSF as a short fiber is effective for microcrack control in HP-HFRCC at both the static rate and high strain rate. However, information on the effect of the blending ratio between long and short steel fibers on the tensile behavior of HP-HFRCC under different strain rates is rather limited. Therefore, in this study, HSF was used as a long fiber and SSF as a short fiber, and the effect of the fiber blending ratio on the tensile behavior of HP-HFRCC was evaluated under different strain rates. In addition, the synergistic effect on the tensile properties of HP-HFRCC was analyzed at the static rate and high strain rate to derive the optimal fiber blending ratio.

Table 1 shows a series of test specimens investigated in this study. First, to compare the performance of HP-HFRCC, a high performance-mono fiber-reinforced cement composite (HP-MFRCC) containing 2.0 vol.% HSF or SSF was fabricated. HP-HFRCC reinforced with HSF and SSF at a ratio of 1.5 + 0.5, 1.0 + 1.0, or 0.5 + 1.5 vol.% was thereafter fabricated.

### 2.2. Materials and Mixture Proportions

Table 2 shows the properties of the materials used in this study and the geometry of the fibers, respectively. For cement, ordinary Portland cement with a density of 3.15 g/cm^3^ and a fineness of 3200 cm^2^/g was used. Fly ash, with a density of 2.20 g/cm^3^ and a fineness of 3200 cm^2^/g, was used as the mineral admixture. In the case of fine aggregates, Type 7 silica sand (maximum particle size: 0.3 mm) with a density of 2.64 g/cm^3^ and an absorptance of 0.38% was used. The particle size distribution of silica sand is shown in Table 3.

**Figure 1:**
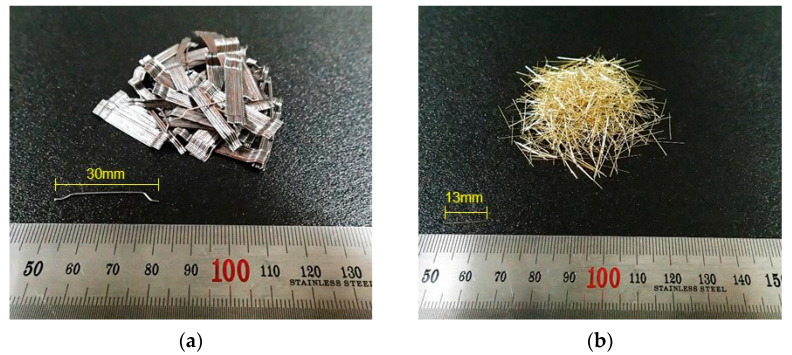
Geometry of the fiber: (**a**) hooked steel fiber (HSF); (**b**) smooth steel fiber (SSF).

In addition, a polycarboxylic acid-based superplasticizer was used to improve the reduce fluidity of the matrix caused by the fiber addition. Figure 1 shows the geometry of the fibers. HSF with a length of 30 mm, diameter of 0.5 mm, density of 7.85 g/cm^3^, and tensile strength of 1140 MPa, and SSF with a length of 12 mm, diameter of 0.2 mm, density of 7.85 g/cm^3^, and tensile strength of 2700 MPa, were used. The HSF featured hook-shaped ends, and the surface of the SSF was coated with brass to prevent corrosion.

Table 4 shows the mix proportions and compressive strength of HPFRCC. The same matrix components as those used in a previous study [37] were used. The water-to-binder (W/B) ratio was set to 0.4, and a small amount of superplasticizer was used to secure the fluidity of the HPFRCC specimen. Fly ash replaced some amount of cement to improve the fluidity of the matrix and dispersibility of the fibers. Silica sand was used as a fine aggregate considering the homogeneity of the matrix and the interfacial adhesion between the fiber and matrix. In addition, fibers were added based on the volume percentage. A twin-shaft mixer was used for the mixing of HPFRCC. Mortar was fabricated by adding mixing water and the superplasticizer after dry mixing the binder and silica sand. A certain amount of fiber was thereafter added at intervals, such that the fibers could be sufficiently dispersed, and it was sufficiently mixed to satisfy the predetermined fluidity. The fluidity of HPFRCC was measured according to ASTM C1437. The flow of H2.0 was 160 mm, and the flow decreased as the volume fraction of SSF increased. Mixed HPFRCC was poured into a mold. The mold filled with HPFRCC was cured for one day in a constant-temperature and constant-humidity chamber with a pouring surface covered with a curing sheet. The mold was thereafter removed after one day, and standard water curing was performed until 28 days of age. The 28-day compressive strength of HPFRCC was provided. All HPFRCC specimens exceeded 60 MPa, and the compressive strengths of H2.0 and S2.0 were 61.06 and 65.98 MPa, respectively. In the case of HP-HFRCC, the compressive strength increased as the volume fraction of HSF decreased, and that of SSF increased because the number of mixed fibers in the matrix increased.

### 2.3. Test Set-up and Procedure

**Figure 2:**
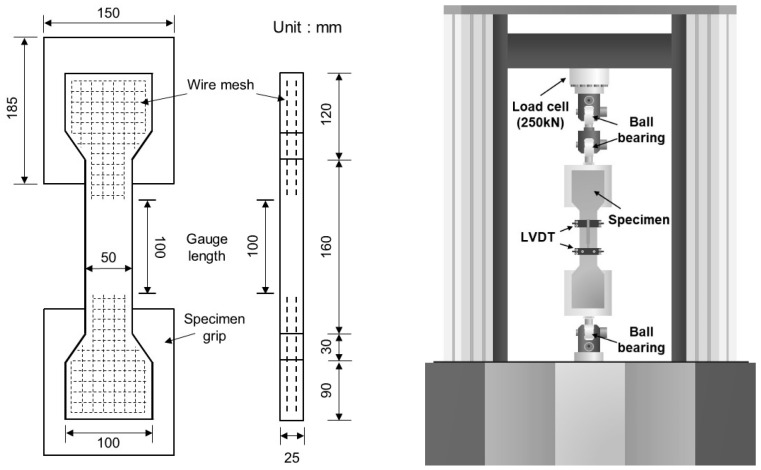
Schematic diagram of direct tensile specimen and static-rate tensile test equipment.

Figure 2 shows the geometry of the tensile specimen evaluated in this study and the static-rate tensile test equipment. The specimen had a length of 400 mm, width of 100 mm, and thickness of 25 mm. It was fabricated in the shape of a dumbbell whose central cross-section decreased to 25 × 50 mm^2^. Each end of the specimen was reinforced with two wire meshes to prevent the fracture of the specimen outside the gauge length. In addition, specimens of the same size were used in both the static-rate tensile test and high-rate tensile test to exclude the size effect. Three specimens per level were evaluated in the static-rate tensile test, and five specimens were evaluated per level in the high-rate tensile test. The static-rate tensile test was conducted using the 250-kN-class static-rate tensile test equipment. The displacement of the specimen was measured by installing linear variable differential transformers (LVDTs) in the front and back of the specimen. The loading rate was set to 1 mm/min, and the average strain rate was measured to be 10^−6^/s.

**Figure 3:**
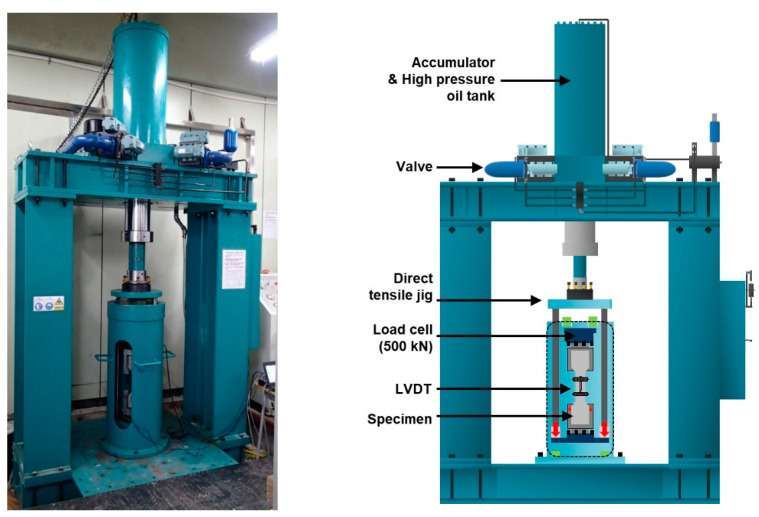
High-rate tensile test equipment.

**Figure 4:**
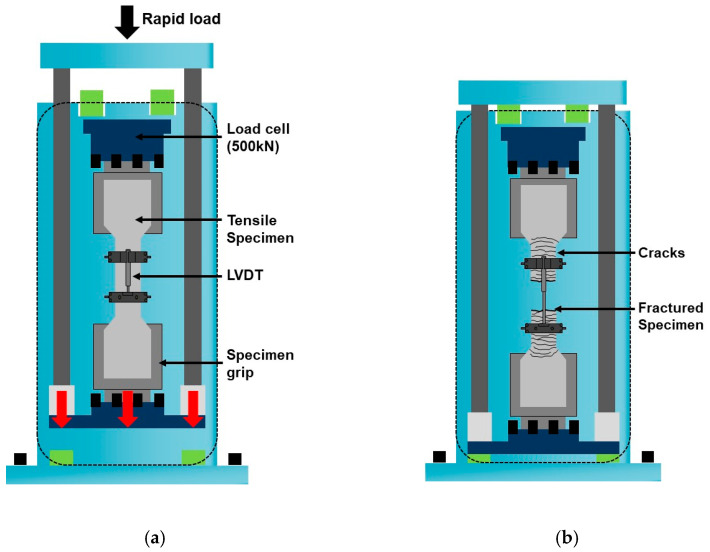
Schematic diagram of the direct tensile jig: (**a**) before the tensile test; (**b**) after the tensile test.

Figure 3 shows the high-rate tensile test equipment. In the equipment, the nitrogen tube is compressed by introducing high-pressure hydraulic oil into the oil tank at the top, which contains the nitrogen tube. The hydraulic pressure is thereafter discharged at once such that the compressed nitrogen tube can expand instantaneously and the generated pressure can be applied as a load at a high rate. The load applied at a high rate was transmitted to the specimen as a tensile load through the direct tensile jig, as shown in Figure 4. The loading rate was set to 5 m/s, and the average strain rate was measured as 10^1^/s. The tensile stress was measured using a load cell installed at the top of the tensile jig. LVDTs were installed in the specimen, and the tensile strain (%) was calculated using the measured displacement (ΔL) and strain measurement range (L). The strain rate was calculated using the slope of the time (t) and strain (ε) curve obtained from each specimen.

**Figure 5:**
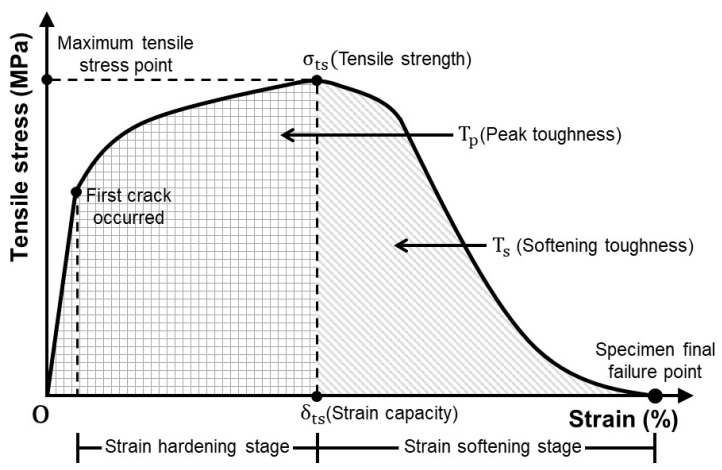
Tensile stress–strain curve and summary of tensile properties.

Figure 5 shows a summary of the tensile properties. For the tensile properties, the maximum stress point of the tensile stress–strain curve obtained by conducting the test was set to the tensile strength ($\sigma_{ts}$), the strain at the tensile strength point to the strain capacity ($\delta_{ts}$), the area under the tensile stress–strain curve up to the tensile strength point to the peak toughness ($T_{p}$), and the area under the tensile stress–strain curve from the tensile strength point to the failure of the specimen to the softening toughness ($T_{s}$).

In addition, in this study, the synergistic effect of the blending ratio between HSF and SSF on the tensile properties was analyzed. The synergistic effect is an index that quantitatively represents the improvement or reduction effect of the tensile properties of HP-HFRCC compared with those of HP-MFRCC, and it was calculated using Equation (1) [31,39].

(1) $$S=\frac{R_{hybrid,a+b}^{\left(V_{f}\right)}-\max\left(R_{mono,a}^{\left(V_{f}\right)},\;R_{mono,\;b}^{\left(V_{f}\right)}\right)}{\max\left(R_{mono,a}^{\left(V_{f}\right)},\;R_{mono,\;b}^{\left(V_{f}\right)}\right)},$$

where $R_{hybrid,\;a+b}$ is the tensile property of HP-HFRCC reinforced with both fibers a and b, $R_{mono,\;a}$ is the tensile property of HP-MFRCC reinforced with fiber a, and $R_{mono,\;b}$ is the tensile property of HP-MFRCC reinforced with fiber b. $R_{hybrid,\;a+b}$, $R_{mono,\;a}$, and $R_{mono,\;b}$ are all based on the same fiber volume fraction ($V_{f}$). Meanwhile, DIF was calculated for each tensile property to analyze the effect of the strain rate on the tensile properties of HPFRCC, according to the fiber blending ratio. DIF was calculated by dividing the value of the tensile property measured through the high-rate tensile test by the value of the tensile property measured through the static-rate tensile test.

## 3. Results and Discussion

**Figure 6:**
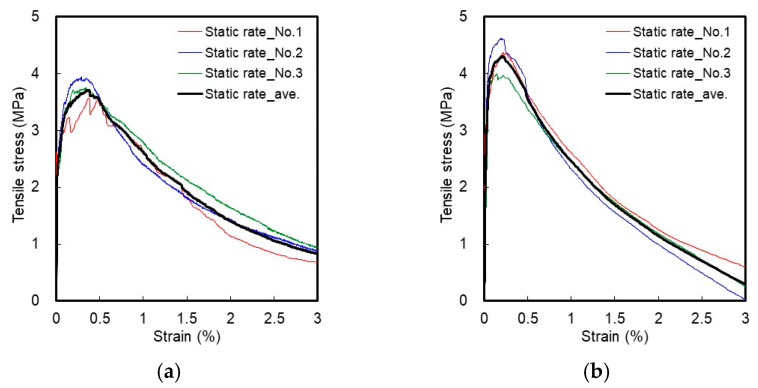

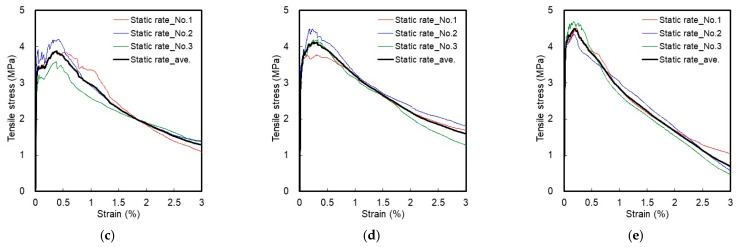
Tensile stress–strain curve at the static rate: (**a**) H2.0; (**b**) S2.0; (**c**) H1.5S0.5; (**d**) H1.0S1.0; (**e**) H0.5S1.5.

**Figure 7:**
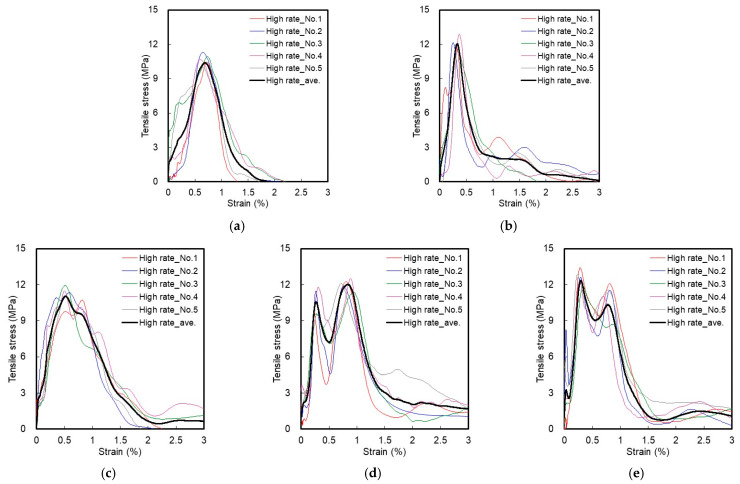
Tensile stress–strain curve at the high rate: (**a**) H2.0; (**b**) S2.0; (**c**) H1.5S0.5; (**d**) H1.0S1.0; (**e**) H0.5S1.5.

This study attempted to analyze the effect of the blending ratio between HSF and SSF on the tensile behavior of HPFRCC under different strain rate conditions. Figure 6 and Figure 7 show the tensile stress–strain curves of HPFRCC according to the static rate (strain rate: 10^−6^/s) and high rate (strain rate: 10^1^/s) tensile loads.

**Figure 8:**
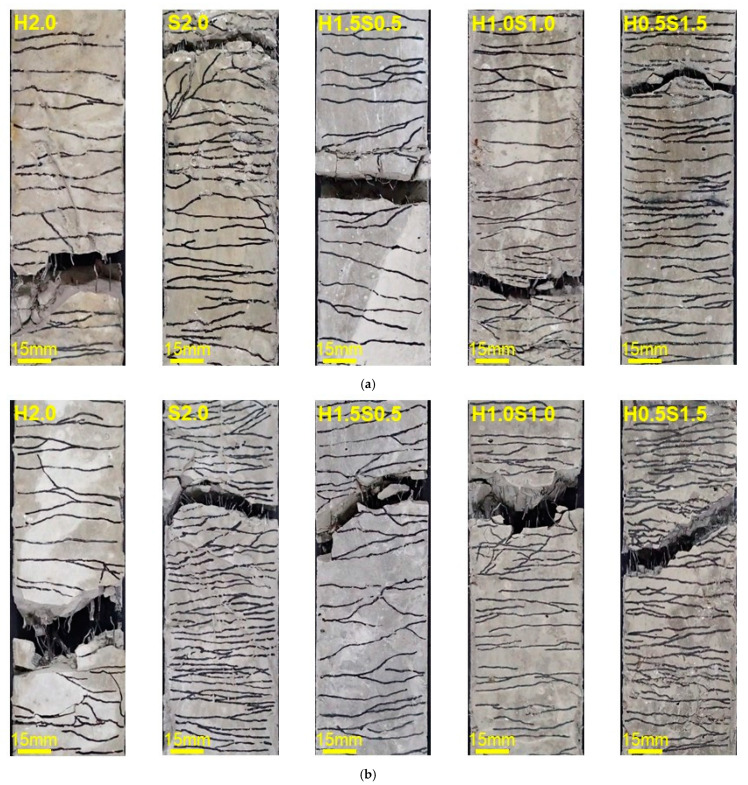
Multiple cracking pattern of HPFRCC within gauge length: (**a**) static rate; (**b**) high rate.

The multiple cracking patterns of the HPFRCC are shown in Figure 8. The tensile properties of HP-MFRCC and HP-HFRCC are summarized in Table 5 and Table 6, respectively.

### 3.1. Static-Rate Tensile Test Result

H2.0 exhibited strain-hardening behavior accompanied with multiple cracks in the matrix due to the bridging effect of HSF after the occurrence of initial cracking at the static rate, and it effectively controlled the macrocracks. In the strain-softening section beyond the tensile strength point, the behavior wherein straightened fibers were pulled out from the matrix, was observed. S2.0 had a smaller diameter and a shorter length compared with HSF and was reinforced with SSF that proved to be effective in controlling microcracks. This increased the number of multiple cracks in S2.0 compared with that in H2.0, owing to a larger number of fibers mixed in the matrix and it exhibited an improvement in the stress during the strain-hardening process. However, it could not effectively control the macrocracks because it had a short fiber length and its end was not deformed. In the strain-softening process, the stress reduction due to the pull-out of the fibers was larger than that in the HSF.

For H1.5S0.5, the number of multiple cracks increased as the SSF controlled the microcracks and HSF the macrocracks, demonstrating the strain-hardening behavior; accordingly, the strain capacity increased in H1.5S0.5 compared with HP-MFRCC [31]. Because the number of fibers mixed in the matrix increased due to the addition of SSF, despite a reduction in the volume fraction of HSF, the tensile strength was higher than that of H2.0. In the strain-softening behavior, the stress reduction was observed to be smaller for H1.5S0.5 compared with that for H2.0, although the volume fraction of HSF decreased. This can be attributed to the fact that the pull-out resistance of HSF was improved by the addition of SSF [40].

H1.0S1.0 also exhibited strain-hardening behavior. Owing to an increase in the volume fraction of SSF, the number of multiple cracks and the tensile strength significantly increased, compared with that in H1.5S0.5. However, the strain capacity decreased. This can be attributed to the fact that the width of multiple cracks decreased as the volume fraction of SSF increased, owing to a significant improvement in the pull-out resistance of HSF [37]. Meanwhile, in the strain-softening behavior, the stress decreased gradually because the HSF with significantly improved pull-out resistance was pulled out from the matrix.

H0.5S1.5 exhibited the largest number of multiple cracks through strain-hardening as well as the highest tensile strength due to HSF, with significantly improved pull-out resistance, although the volume fraction of SSF was lower than that of S2.0. However, the strain capacity was found to be the lowest. This can be attributed to the fact that the width of multiple cracks decreased because the volume fraction of HSF that controls macrocracks is low (0.5 vol.%) and the pull-out resistance is significantly improved. Nonetheless, the HSF, with significantly improved pull-out resistance, caused a significant friction in the pull-out process from the matrix beyond the tensile strength point. Therefore, the stress reduction in the strain-softening behavior of H0.5S1.5 was smaller than that in S2.0.

### 3.2. High-Rate Tensile Test Result

As the strain rate increased under high-rate tensile loading, the bonding strength between the fiber and matrix increased, and most tensile properties of HP-MFRCC significantly increased compared with the static rate. For H2.0, the number of multiple cracks, tensile strength, and strain capacity significantly increased, compared with the static rate, and most of the HSFs were observed to be non-straightened. For S2.0, all the tensile properties were improved compared with the static rate, owing to an increase in the strain rate. However, it was observed that the macrocracks were not effectively controlled after regulating the microcracks due to the short fiber length. The SSF was pulled out from the matrix in the strain-softening stage, resulting in a relatively rapid stress reduction. Unlike H2.0, which exhibited the final failure (that is, zero stress) before the strain reached 2% due to the destruction of the matrix caused by insufficient friction between the fiber and the matrix, S2.0 had several specimens that maintained stress even when the strain exceeded 3%. 

For HP-HFRCC, unlike HP-MFRCC, all the tensile properties significantly increased owing to an increase in the strain rate. It also exhibited tensile behavior that was different from the static-rate tensile behavior as the bonding strength between the fiber and matrix increased under high-rate tensile loading. For H1.5S0.5, the number of multiple cracks, tensile strength, and strain capacity increased compared with the static rate, owing to an increase in the strain rate. While the strain capacity in H1.5S0.5 was higher than that in H2.0 at the static rate, it exhibited a lower strain capacity at a high strain rate. In the strain-softening section, the number of HSFs that were pulled out in a straightened form increased compared with that in H2.0, and the sharp reduction in stress was countered to a certain extent.

Furthermore, there were two peak stress points in the tensile stress–strain curves of H1.0S1.0 and H0.5S1.5 under high-rate tensile loading. In the case of H1.0S1.0, the tensile strength (that is, the point reached by the maximum stress) was formed at the second peak stress point. For H0.5S1.5, the tensile strength was formed at the first peak stress point. This difference in the tensile behavior could be attributed to the fiber blending ratio between HSF and SSF. For H1.0S1.0, after the occurrence of initial cracking, the first peak stress point was formed while SSF was controlling the microcracks, and the second peak stress point was formed as HSF was controlling the macrocracks. It may be considered that the second peak stress point was higher than the first peak stress point because the pull-out resistance of HSF was significantly improved. The improvement in the pull-out resistance of HSF can be considered to have improved the sharp stress reduction phenomenon in the strain-softening section beyond the tensile strength point and significantly increased the number of HSFs that were pulled out in a straightened form.

H0.5S1.5 also exhibited two peak stress points; however, the first peak stress point was higher than the second peak stress point. Owing to an increase in the volume fraction of SSF, the stress improvement of the first peak point was higher in H0.5S1.5 than that in H1.0S1.0; however, the stress improvement of the second peak point was lower, although the pull-out resistance of HSF was significantly improved owing to a reduction in the volume fraction of HSF. In addition, it appears that the stress reduction after the second peak point was larger than that after H1.0S1.0 owing to the low volume fraction of HSF that was effective for the control of the macrocracks.

### 3.3. Fiber Failure Behavior under Different Strain Rates

In general, the bonding mechanisms between the steel fiber and matrix are divided into three types: adhesive bonding, mechanical bonding, and friction bonding [41]. HSF is dominated by mechanical bonding, whereas SSF is mainly affected by adhesive and friction bonding [31]. An increase in the strain rate improves the interfacial adhesive bonding, mechanical bonding, and friction bonding between the fiber and matrix; nonetheless, the changes in the tensile properties of H2.0 and S2.0, owing to the increase in strain rate, were significantly different. This can be attributed to the fact that the influence of the strain rate was different depending on the bonding mechanism between the fiber that reinforced the tensile properties of HPFRCC and the matrix, as well as the fiber failure behavior. Therefore, the fiber failure behavior, with respect to the strain rate, was analyzed.

At the static rate, HSF causes micro-split cracks in the surrounding matrix owing to its hook-shaped ends, while it controls the cracks generated in the specimen [42,43]. Such micro-split cracks are generated because a high local compression pressure is formed between the fiber and matrix around the hook-shaped ends (that is, parts where the fiber was bent) of HSF. The micro-split cracks generated in the matrix around the HSF affect the stress reduction in the fiber pull-out process; however, the fibers are pulled out in a straightened form owing to the friction against the matrix. At a high strain rate, the adhesive and mechanical bonding of HSF increases, and the tensile properties (tensile strength, strain capacity, and peak toughness), in particular, are significantly improved owing to the mechanical bonding and high strain rate sensitivity [29]. An increase in the strain rate, however, increases the micro-split cracks that are generated in the matrix around the HSF [37]. Beyond the tensile strength point, the increased micro-split cracks form the matrix in fragments, which causes most HSFs to remain non-straightened. Because of the change in the failure behavior of HSF caused by the strain rate, the softening toughness value of H2.0 at a high strain rate was lower than the value at the static rate.

Unlike HSF, which is dominated by mechanical bonding, SSF is mainly affected by adhesive and friction bonding. It is known that the friction bonding of SSF demonstrates a negligible strain rate sensitivity at a normal strength (W/C = 0.5) [44]; however, this result has been obtained for the case wherein fine aggregate of the river sand grade was used as a matrix component. According to Wille et al. [45], the use of silica sand (maximum diameter: 0.8 mm) in UHPFRC causes scratches on the brass coat on the surface of steel fiber that may improve friction bonding. 

The HPFRCC in this study did not exhibit ultra-high strength ($f_{c}$ = approximately 200 MPa), but it used Type 7 silica sand (maximum diameter: 0.3 mm) as a matrix component. In addition, because SSF—the surface of which was coated with brass—was used (Figure 1), an improvement in the friction bonding of SSF under the high-rate tensile loading was expected. Furthermore, the SSF used in this study had a short length (13 mm) and small diameter (0.2 mm); therefore, a significant improvement in adhesive bonding was expected owing to the large adhesive area between the fiber and matrix [39]. As the strain rate increased, the tensile properties (tensile strength, strain capacity, and peak toughness) of S2.0 improved; however, the DIF values of S2.0 were not higher than those of H2.0 (Table 5). Moreover, the softening toughness value of S2.0 at the high strain rate was barely improved compared with the value at the static rate. This was different from the expected results, implying that the increase in the strain rate barely improved the friction bonding of SSF and did not significantly improve the adhesive bonding.

**Figure 9:**
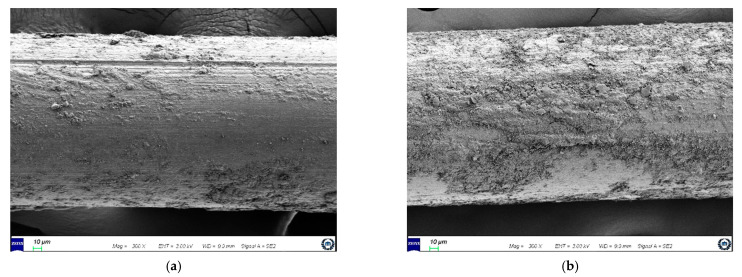
Surface condition of SSF after high-rate tensile test (SEM image): (**a**) case with scratched on the surface; (**b**) case with mortar attached on the surface.

It can be guessed that the adhesive bonding of the SSF that was used in S2.0 was not significantly improved owing to a reduction in the bonding efficiency caused by the porosity of the fiber–matrix interface [46] and a reduction in the bonding performance caused by the low embedded depth of the fibers based on the cracking area [47]. However, these reasons are not sufficient to support the finding that the friction bonding of SSF was barely improved. To identify the exact causes, the surface of the SSF in the fracture section of S2.0, upon completion of the high-rate tensile test, was analyzed using a scanning electron microscope (SEM), as shown in Figure 9. 

The surface condition of SSF was categorized into two cases: a case wherein few scratches were generated and a small part of the matrix was left on the surface, and another case wherein no scratch was generated and the matrix was attached to the large part of the surface. This is an extremely interesting result, and it should be noted that the matrix was attached and left on the surface although the SSF was pulled out from the matrix. In general, when the SSF is pulled out from the matrix, the interface attached to the matrix is detached, and the friction between the fiber surface and matrix is experienced during the pull-out process [43]. However, after the high-rate tensile test conducted in this study, the matrix was still attached to the surface of the SSF, despite the pull-out of SSF. According to Tran et al. [6], when a mortar-based HPFRCC is subjected to high-rate tensile loading, additional clamping pressure occurs on the fiber surface owing to the occurrence of the inertia effect in the matrix around the steel fiber, thereby increasing the interfacial adhesive bonding between the fiber and matrix. Through this process, they reported that the post-cracking strength significantly increased; however, the initial cracking strength (that is, the tensile strength of the mortar-based matrix) did not increase significantly at the high strain rate. Therefore, for the SSF of S2.0 subjected to high-rate tensile loading in this study, it seems that detachment did not occur at the fiber–matrix interface, and the matrix around the fiber was fractured because the tensile strength of the matrix around the fiber was not significantly improved, although the interfacial adhesive bonding between the fiber and matrix was significantly improved. The DIFs of the tensile properties (tensile strength, strain capacity, and peak toughness) of S2.0 were lower than those of H2.0 because the matrix around the fiber was fractured before the occurrence of detachment at the SSF–matrix interface.

Further, it was observed that the softening toughness value of S2.0 at the high strain rate was barely improved, compared with the static rate, mainly due to the short length of the SSF. When the specimen under high-rate tensile loading is fractured, it is highly probable that the SSF with a short fiber length that is exposed to the fracture section is pulled out, without friction on the surface (Figure 9b). For SSF with a long fiber length that is exposed to the fracture section, friction is generated on the surface; however, it is estimated that most of the friction occurs on the matrix attached to the surface, and the friction that may cause scratches on the fiber surface is limited (Figure 9a). Consequently, the matrix components of HPFRCC used in this study satisfied the conditions for the improvement of the friction bonding of SSF at the high strain rate; nonetheless, it is concluded that friction bonding is barely improved, owing to the short fiber length and the change in the fiber failure behavior.

### 3.4. Hybrid Effect of Steel Fibers in HPFRCC

When either HSF or SSF was used in HPFRCC, they exhibited different failure behaviors according to the strain rate. Therefore, hybrid reinforcement with HSF and SSF affects the failure behavior of each fiber at different strain rates. It is expected that not only the strain rate, but also the fiber blending ratio, will affect the failure behavior of each fiber. In this study, the tensile behavior of HP-HFRCC was analyzed to identify the effects of the strain rate and fiber blending ratio on the failure behavior of each fiber. When compared with H2.0 at the static rate, H1.5S0.5 exhibited an increase in the strain capacity as SSF controlled the microcracks and HSF the macrocracks. The addition of SSF increased the tensile strength because it increased the number of fibers mixed in the matrix and improved the pull-out resistance of HSF. In addition, despite a reduction in the volume fraction of HSF, the stress reduction in the strain-softening process was smaller, owing to an improvement in the pull-out resistance of HSF. 

**Figure 10:**
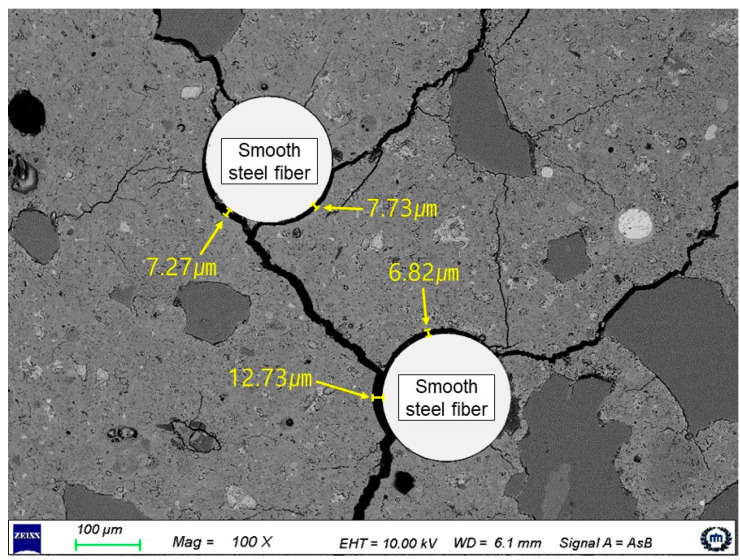
Interfacial gap between the matrix and SSF by the micro split cracks at high strain rates (HP-HFRCC).

When compared with H2.0 at a high strain rate, H1.5S0.5 exhibited a reduction in strain capacity, unlike the static rate. To identify the cause of the reduction in strain capacity, the SEM-backscattered electron image was analyzed for the cross section of HP-HFRCC, after conducting the high-rate tensile test, as shown in Figure 10. It should be noted that gaps were formed by cracking at the interface between a few SSFs around HSF and the matrix. At the high strain rate, HSF causes many micro-split cracks in the surrounding matrix [37]. Therefore, it could be confirmed that the micro-split cracks generated by the HSF affected the interface between the surrounding SSF and matrix. In addition, the results of the softening toughness values of 9.15 and 9.74 Nm at the static rate and high strain rate, respectively, indicate that 0.5 vol.% SSF is not sufficient to effectively improve the pull-out resistance of HSF at the high strain rate. In summary, while SSF was controlling the microcracks in the specimen, micro-split cracks were generated in the matrix by HSF, and the formation of macrocracks was accelerated as gaps were formed at the interface between a few SSFs and the matrix. Because the pull-out resistance of HSF, which controlled the macrocracks, was not sufficiently improved, it could be considered that the strain-softening section, where the stress decreased, was formed relatively rapidly.

For H1.0S1.0, each fiber effectively controlled the microcracks and macrocracks at the static rate; nonetheless, unlike H1.5S0.5, the strain capacity decreased. This is because the width of multiple cracks decreased as the SSF with an increased volume fraction significantly improved the pull-out resistance of the HSF. Owing to the significantly improved pull-out resistance of HSF, H1.0S1.0 exhibited the smallest stress reduction in the strain-softening section. At a high strain rate, H1.0S1.0 exhibited tensile behavior wherein two peak stress points were formed. This appears to be related to the influence of the micro-split cracks in the matrix caused by the HSF on the SSF–matrix interface. Under high-rate tensile loading, SSF controls the microcracks of H1.0S1.0, thereby increasing the initial stress. Subsequently, gaps were generated at the interface between a few SSFs that controlled the microcracks in the specimen and the matrix due to the micro-split cracks in the matrix around the HSF, and this caused the stress reduction (formation of the first peak stress point). Unlike H1.5S0.5, however, the stress increased again as HSF with significantly improved pull-out resistance effectively controlled the macrocracks (formation of the second peak stress point). As the pull-out resistance of HSF was significantly improved, the stress was observed to have decreased gradually in the strain-softening process beyond the second peak stress point. Consequently, when the fibers whose failure behavior varies depending on the strain rate are mixed at an appropriate ratio, they are expected to produce a synergy that has a positive influence on the tensile behavior.

H0.5S1.5 effectively controlled the microcracks at the static rate, but nonetheless exhibited a strain capacity that was lower than that of S2.0 because the width of multiple cracks was reduced by HSF with significantly improved pull-out resistance. In addition, the stress reduction in the strain-softening process was improved to some extent compared with S2.0 because HSF controlled the macrocracks; however, the stress reduction in H0.5S1.5 occurred relatively rapidly compared with that in H1.0S1.0. At the high strain rate, tensile behavior was observed wherein two peak stress points were formed; nonetheless, the stress of the first peak stress point was higher, unlike that in H1.0S1.0. The stress did not significantly increase, compared with the first peak stress point, owing to the low volume fraction of HSF, although the HSF, with significantly improved pull-out resistance, controlled the macrocracks after the formation of the first peak stress point. In addition, the stress rapidly decreased after the second peak stress point, owing to the low volume fraction of HSF.

**Figure 11:**
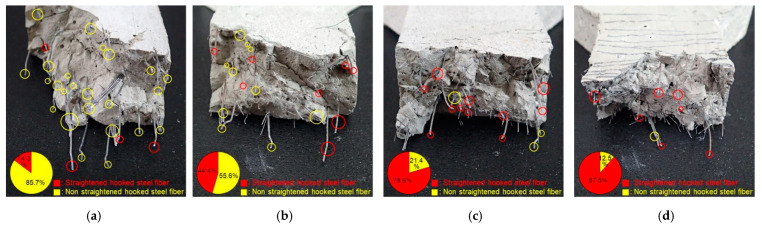
Fiber end-state of HSF in the fracture section after high-rate tensile test: (**a**) H2.0; (**b**) H1.5S0.5; (**c**) H1.0S1.0; (**d**) H0.5S1.5.

Figure 11 shows the end-state of the HSF in the fracture section after the high-rate tensile test was conducted. At a high strain rate, the HSF of H2.0 increased the micro-split cracks in the surrounding matrix owing to its hook-shaped ends, and most of the fibers remained non-straightened (Figure 11a). For H1.5S0.5, the number of HSFs pulled out in a straightened form increased; however, it did not exceed half of the total number of HSFs (Figure 11b). This confirmed that the 0.5 vol.% SSF was not sufficient to significantly improve the pull-out resistance of HSF at the high strain rate. For H1.0S1.0 and H0.5S1.5, the number of HSFs pulled out in a straightened form compared with the total number of HSFs was 78.6% and 87.5%, respectively. These results indicate that the SSF of 1.0 vol.% more effectively controlled the micro-split cracks generated in the matrix around HSF, thereby significantly improving the pull-out resistance of HSF. Based on this, it was confirmed that the blending ratios of microfibers and macrofibers have a significant influence on the failure behavior of each other and may also significantly impact the tensile behavior of HP-HFRCC.

### 3.5. Synergistic Effect of Fiber Blending Ratio on the Tensile Properties

**Figure 12:**
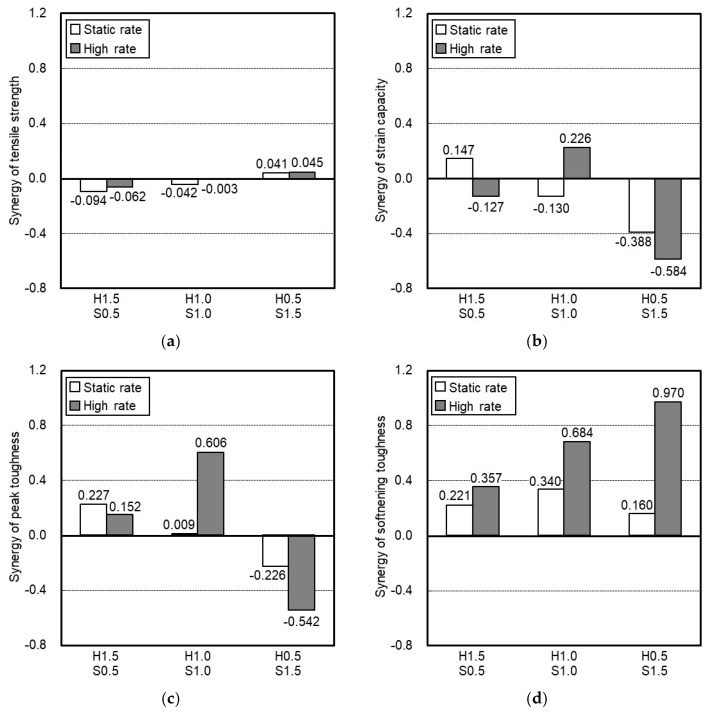
Synergistic effect on the tensile properties of HP-HFRCC: (**a**) tensile strength; (**b**) strain capacity; (**c**) peak toughness; (**d**) softening toughness.

Figure 12 shows the synergistic effect on the tensile strength, strain capacity, peak toughness, and softening toughness of HP-HFRCC according to the fiber blending ratio. The blending of HSF and SSF did not have a significant influence on the synergy of the tensile strength of HP-HFRCC, compared with the other tensile properties. It appears that the synergy of tensile strength is significantly affected by an increase in the number of fibers mixed in the matrix and an improvement in the pull-out resistance of HSF caused by the addition of SSF. At the static rate, H0.5S1.5 exhibited the highest synergy of tensile strength (0.041), followed by H1.0S1.0 (−0.042) and H1.5S0.5 (−0.094). This implies that an increase in the number of fibers mixed in the matrix has a more significant influence on the synergy of the tensile strength at the static rate.

Similarly, at a high strain rate, H0.5S1.5 exhibited the highest synergy of tensile strength (0.045), followed by H1.0S1.0 (−0.003) and H1.5S0.5 (−0.062). Compared with the static rate, however, H1.5S0.5 and H1.0S1.0 exhibited increases in the synergy of the tensile strength by 0.032 and 0.039, respectively. H0.5S1.5 did not show a significant increase in the synergy of tensile strength (0.004). For H1.5S0.5 and H1.0S1.0, the synergy of tensile strength increased compared with the static rate, owing to an improvement in the pull-out resistance of HSF at a high strain rate, and H1.0S1.0, with significantly improved pullout resistance, exhibited a higher rate of increase in synergy. For H0.5S1.5, however, the synergy of tensile strength did not significantly improve because the volume fraction of HSF decreased. This indicates that the influence of an improvement in the pull-out resistance of HSF significantly increases the synergy of tensile strength at the high strain rate.

In the case of the synergy of strain capacity, different results were obtained depending on the fiber blending ratio and the strain rate. At the static rate, H1.5S0.5 exhibited the highest synergy of strain capacity (0.147), followed by H1.0S1.0 (−0.130) and H0.5S1.5 (−0.388). As the volume fraction of SSF increased, the synergy of the strain capacity of HP-HFRCC decreased. For all HP-HFRCC specimens, at the static rate, SSF controlled the microcracks and HSF the macrocracks. However, when the volume fraction of SSF exceeded 1.0 vol.%, the pull-out resistance of HSF was significantly improved, resulting in a reduction in the width of multiple cracks (Table 5 and Table 6). Moreover, for H0.5S1.5, the synergy of the strain capacity significantly decreased owing to the low volume fraction of HSF that controlled the macrocracks. However, different results were obtained at the high strain rate. The synergy of the strain capacity of H1.5S0.5 decreased to −0.127, and H1.0S1.0 exhibited the highest synergy of strain capacity (0.226). For H1.5S0.5, the pull-out resistance of HSF was not significantly improved, and therefore, HSF could not effectively control the macrocracks. In the case of H1.0S1.0, however, the highest synergy of strain capacity was observed because 1.0 vol.% SSF significantly improved the pull-out resistance of HSF and HSF effectively controlled the macrocracks. For H0.5S1.5, SSF significantly improved the pull-out resistance of HSF; however, the synergy of the strain capacity decreased compared with the static rate, owing to the low volume fraction of HSF.

At the static rate, H1.5S0.5 exhibited the highest synergy of peak toughness (0.227). This is due to the effective control of micro- and macrocracks by each fiber. For H1.0S1.0, micro- and macrocracks were effectively controlled; however, the synergy (0.009) was not high because the width of multiple cracks decreased owing to an improvement in the pull-out resistance of the HSF. For H0.5S1.5, a negative synergy (−0.226) was observed owing to the reduction in the macrocrack control capacity and the width of multiple cracks caused by the low volume fraction of HSF. At the high strain rate, H1.0S1.0 exhibited the highest synergy of peak toughness (0.606). This is because a high energy absorption capacity was achieved as SSF controlled the microcracks, and the HSF, with significantly improved pull-out resistance, controlled the macrocracks. Meanwhile, it should be noted that H1.5S0.5 exhibited a positive synergy of peak toughness (0.152) at a high strain rate, while it exhibited a negative synergy of both tensile strength (−0.062) and strain capacity (−0.127). This can be attributed to the fact that it exhibited a relatively high energy absorption capacity as the SSF controlled the microcracks, and the HSF, with partially improved pull-out resistance (Figure 11b), controlled the macrocracks.

For all HP-HFRCC specimens, the synergy of the softening toughness was found to be positive. In particular, the synergy significantly increased at a high strain rate. At the static rate, H1.0S1.0 exhibited the highest synergy of softening toughness (0.340). This is because it had the largest number of HSFs with significantly improved pull-out resistance. The synergy of the softening toughness of H1.5S0.5 was 0.221. This is because the pull-out resistance of HSF was not significantly improved owing to the low volume fraction of SSF, despite having the largest number of HSFs among the HP-HFRCC specimens. H0.5S1.5 exhibited the lowest synergy (0.160) owing to the low volume fraction of HSF, although the pull-out resistance of HSF was significantly improved. Meanwhile, at a high strain rate, H0.5S1.5 exhibited the highest synergy of softening toughness (0.970). This is because a high fracture energy absorption capacity was achieved after the tensile strength point due to the HSF with significantly improved pull-out resistance, despite its low volume fraction (0.5 vol.%). H1.0S1.0 exhibited a lower synergy (0.684) than H0.5S1.5, although HSF with significantly improved pull-out resistance also absorbed considerable fracture energy, because it was already involved in absorbing some amount of energy before the complete failure (strain-softening stage) of the specimen began. H1.5S0.5 exhibited a positive synergy of softening toughness (0.357); however, it was the lowest because the pull-out resistance of HSF was not significantly improved.

### 3.6. Strain Rate Sensitivity of the Tensile Properties

**Figure 13:**
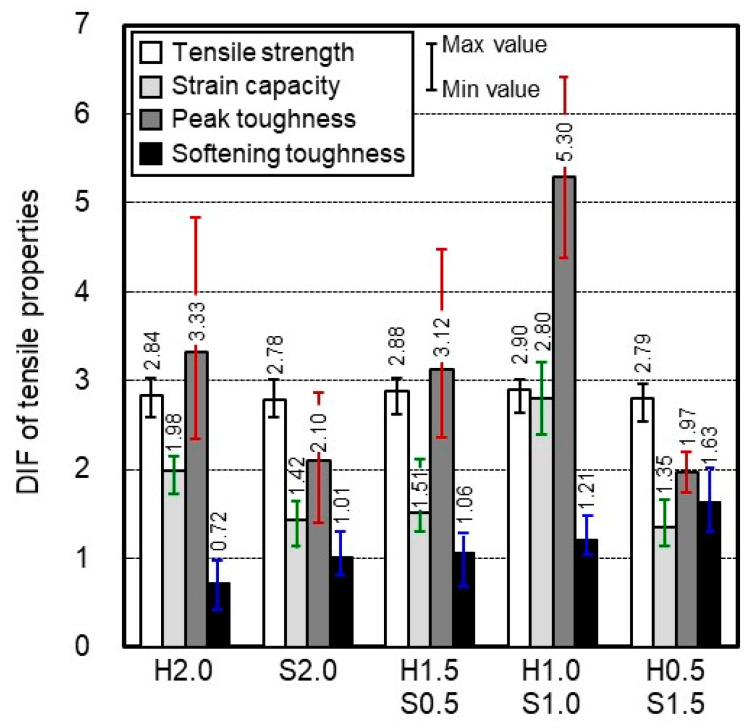
Effect of strain rate on the tensile properties of HPFRCC with different fiber blending ratios.

Figure 13 shows the effect of strain rate on the tensile properties of HPFRCC with different fiber blending ratios. It was found that the DIF of tensile strength (a minimum of 2.78, and a maximum of 2.90) was not significantly affected by the fiber blending ratio compared with the DIFs of the other tensile properties. For a matrix with a high strength, the DIF of tensile strength may decrease when a few of the long steel fibers are replaced with short steel fibers [31]. In this study, however, the strength level did not have a significant influence on shrinkage; therefore, the impact of long fibers on interfacial adhesive bonding was insignificant. Therefore, few HP-HFRCC specimens exhibited higher values for the DIF of tensile strength than HP-MFRCC. The DIFs of the strain capacity and peak toughness varied significantly depending on the fiber blending ratio. In particular, it was found that the DIF of peak toughness was the most affected (a minimum of 1.97 and a maximum of 5.30). In the case of the DIF of softening toughness, HP-HFRCC exhibited a higher strain rate sensitivity than HP-MFRCC. 

Among the HPFRCC specimens evaluated in this study, H1.0S1.0 exhibited the highest DIFs of tensile strength, strain capacity, and peak toughness, with the values of 2.90, 2.80, and 5.30, respectively. This appears to be due to the synergistic effect caused by the microcrack control of SSF and the macrocrack control of HSF, with significantly improved pull-out resistance. The DIF of the softening toughness was 1.21. Because HSF with significantly improved pull-out resistance was involved in absorbing some amount of energy before the strain-softening stage, the highest strain rate sensitivity was not observed (1.63 for H0.5S1.5). However, considering that the values of H2.0, S2.0, and H1.5S0.5 for the DIF of softening toughness were 0.72, 1.01, and 1.06, respectively, the strain rate sensitivity of H1.0S1.0 for softening toughness was not low per se.

The DIFs of tensile properties were compared between H2.0 and H1.5S0.5 to evaluate the impact of replacing a few HSFs with SSFs on the strain rate sensitivity of tensile properties. The DIF of the tensile strength of H1.5S0.5 (2.88) was higher than that of H2.0 (2.84). This is because the number of fibers mixed in the matrix increased, and the pull-out resistance of the HSF was partially improved. At a high strain rate, however, the addition of SSF accelerated the generation of macrocracks in the specimen (Figure 10), and the HSF with partially improved pull-out resistance could not effectively control the macrocracks. As a result, H1.5S0.5 exhibited lower values for the DIFs of strain capacity and peak toughness than H2.0. However, it exhibited a higher value for the DIF of softening toughness because the pull-out resistance of HSF was partially improved and the number of straightened HSFs increased at the high strain rate (0.72 and 1.06 for H2.0 and H1.5S0.5, respectively).

The DIFs of tensile properties were compared between S2.0 and H0.5S1.5 to evaluate the impact of replacing a few SSFs with HSFs on the strain rate sensitivity of tensile properties. The values of S2.0 and H0.5S1.5 for the DIF of tensile strength were 2.78 and 2.79, respectively, indicating no significant difference in the strain rate sensitivity. Replacing a few SSFs with HSFs decreased the DIFs of strain capacity and peak toughness because the HSF with improved pull-out resistance reduced the width of multiple cracks of H0.5S1.5 (11.68 and 9.72 µm for S2.0 and H0.5S1.5, respectively). The DIF of the softening toughness of H0.5S1.5 (1.63), however, was much higher than that of S2.0 (1.01). This is because the fracture energy was effectively absorbed as the pull-out resistance of the HSF was significantly improved. Consequently, replacing a few HSFs with SSFs, or a few SSFs with HSFs (0.5 vol.%), did not have a significant effect on the improvement of the strain rate sensitivity of tensile strength, and was negative for the strain rate sensitivity of strain capacity and peak toughness. However, it was highly effective in improving the strain rate sensitivity of the softening toughness.

## 4. Conclusions

This study analyzed the synergistic effect of the blending ratio of HSFs and SSFs on the tensile properties of HPFRCCs under different strain rate conditions. To analyze the synergistic effect, H2.0 and S2.0 specimens reinforced with 2.0 vol.% HSF and SSF, respectively, were prepared. A HP-HFRCC specimen was fabricated by mixing HSF and SSF at the ratios of 1.5 + 0.5, 1.0 + 1.0, and 0.5 + 1.5 vol.%, and direct tensile tests were conducted at a static rate (strain rate: 10^−6^/s) and high rate (strain rate: 10^1^/s). The following conclusions can be drawn:

1. The SSF was pulled out with friction between the fiber surface and matrix after the detachment of the fiber–matrix interface at the static rate. When the strain rate increased, the interfacial adhesive bonding between the fiber and matrix increased, resulting in the destruction of the matrix around the fiber, without the detachment of the fiber–matrix interface. This caused different pull-out behaviors depending on the length of the fibers exposed at the fracture section of the specimen. When the exposed length of the fiber was long, only a few scratches appeared on the fiber surface owing to the friction between the matrix attached to the fiber surface and the matrix of the cementitious composite. On the other hand, when the length of the exposed fiber was short, it was confirmed that the matrix remained on the fiber surface.
2. At the static rate, in the case of reinforcement with both HSF and SSF, the pull-out resistance of HSF was improved as SSF controlled the micro-split cracks generated in the matrix around the HSF. When the volume fractions of SSF were 1.0% and 1.5%, the width of multiple cracks in the specimen was reduced. At a high strain rate, microcracks were controlled by SSF, whereas micro-split cracks were generated in large quantities in the matrix around the HSF. In this instance, the micro-split cracks formed gaps at the interface between a few SSFs and the matrix, thereby accelerating the generation of macrocracks. It was confirmed that the micro- and macrocrack control capacities varied depending on the blending ratio between HSF and SSF. Therefore, it is necessary to derive an appropriate blending ratio between HSF and SSF.
3. H1.5S0.5 exhibited positive synergy of the tensile properties (strain capacity, peak toughness, and softening toughness) at the static rate owing to the control of the micro- and macrocracks. At a high strain rate, however, SSF with a low volume fraction (0.5%) could not significantly improve the pull-out resistance of HSF. Therefore, HSF was unable to effectively control the macrocracks, thereby significantly decreasing the strain capacity and peak toughness synergy.
4. At both the static rate and high strain rate, H0.5S1.5 exhibited positive synergy of the tensile strength and softening toughness, owing to the large number of SSFs in the matrix and the significantly improved pull-out resistance of HSF. Furthermore, negative synergy was observed in the strain capacity and peak toughness because the width of multiple cracks in the specimen was reduced owing to the improvement in the pull-out resistance of the HSF.
5. At the static rate, H1.0S1.0 exhibited positive synergy of the energy absorption capacity (peak toughness and softening toughness) because each fiber effectively controlled the micro- and macrocracks. At a high strain rate, the synergy increased in all the tensile properties because HSF with significantly improved pull-out resistance effectively controlled the macrocracks. Consequently, H1.0S1.0 exhibited higher strain rate sensitivities of tensile properties than HP-MFRCC (H2.0 and S2.0) and exhibited the highest DIFs of tensile strength, strain capacity, and peak toughness among the HPFRCC specimens evaluated in this study.

It was confirmed that the fiber blending ratio that shows a synergistic effect of high tensile properties varies depending on the reinforcing fiber type. In particular, in the case of the DIF of tensile properties, the blending of 1.0% HSF and 1.0% SSF was most effective in this study, but the blending of 1.5% HSF and 0.5% PVA was most effective in a previous study [37]. Based on the results and conclusions, future research should investigate the resistance of HP-HFRCC to higher strain rates. In particular, the impact and explosion resistance for HP-HFRCC, which exhibited high DIFs of tensile properties such as H1.0S1.0, should be evaluated.

## Figures and Tables

**Table 1:** Series of test specimens.

Type	ID.	Fiber Volume Fraction (vol.%)	
		Hooked Steel Fiber (HSF)	Smooth Steel Fiber (SSF)
HP-MFRCC ^a^	H2.0	2.0	
	S2.0		2.0
HP-HFRCC ^b^	H1.5S0.5	1.5	0.5
	H1.0S1.0	1.0	1.0
	H0.5S1.5	0.5	1.5

^a^ High-performance mono fiber-reinforced cementitious composite. ^b^ High-performance hybrid fiber-reinforced cementitious composite.

**Table 2:** Mechanical properties of the used materials.

Materials	Mechanical Properties
Cement	Ordinary Portland cement; Density: 3.15 g/cm^3^; Fineness: 3200 cm^2^/g
Fly ash	Density: 2.20 g/cm^3^; Fineness: 3000 cm^2^/g
Silica sand	Type 7; Density: 2.64 g/cm^3^; Absorptance: 0.38%
Super plasticizer	Polycarboxylic acid type; specific gravity: 1.05 ± 0.05; pH: 5.0 ± 1.5
Hooked steel fiber (HSF)	Length: 30 mm; Diameter: 0.5 mm; Density: 7.85 g/cm^3^ Tensile strength: 1140 MPa; Elastic modulus: 200 GPa
Smooth steel fiber (SSF)	Length: 13 mm; Diameter: 0.2 mm; Density: 7.85 g/cm^3^ Tensile strength: 2700 MPa; Elastic modulus: 200 GPa

**Table 3:** Silica sand particle size distribution.

Silica Sand (Type 7)	Sieve Size					
	0.3 mm (No.50)	0.212 mm (No.70)	0.15 mm (No.100)	0.106 mm (No.140)	0.075 mm (No.200)	0.053 mm (No.270)
Passing sieve (%)	0.3	41.3	49.1	7.7	1.2	0.4

**Table 4:** Mix proportions and compressive strength of HPFRCC.

ID.	W/B	Unit Weight (kg/m^3^)				Flow (mm)	Compressive Strength (MPa)
		Water	Cement	Fly Ash	Silica Sand		
H2.0	0.4	0.4	0.85	0.15	0.35	160	61.06
S2.0						130	65.98
H1.5S0.5						155	61.80
H1.0S1.0						140	62.21
H0.5S1.5						130	64.92

**Table 5:** Tensile properties of HP-MFRCC.

ID.	Strain Rate	Specimen No.	$\mathrm{Tensile}\;\mathrm{Strength}\;(\sigma_{ts})$		$\mathrm{Strain}\;\mathrm{Capacity}\;(\delta_{ts})$		$\mathrm{Peak}\;\mathrm{Toughness}\;(T_{p})$		$\mathrm{Softening}\;\mathrm{Toughness}\;(T_{s})$		Number of Cracks	Average Crack Width
	/s		MPa	DIF	%	DIF	N·m	DIF	N·m	DIF	EA	µm
H2.0	0.000001	No.1	3.56	-	0.38	-	1.55	-	6.72	-	11	34.55
	0.000001	No.2	3.94	-	0.29	-	1.32	-	7.61	-	7	41.43
	0.000001	No.3	3.75	-	0.34	-	1.46	-	8.14	-	7	48.57
	**0.000001**	**Average**	**3.75**	**-**	**0.34**	**-**	**1.44**	**-**	**7.49**	**-**	**8.3**	**40.96**
	12.23	No.1	10.43	2.78	0.72	2.14	4.17	2.89	3.13	0.42	13	55.39
	19.20	No.2	11.28	3.01	0.66	1.93	3.46	2.40	5.76	0.77	12	55.00
	12.93	No.3	10.92	2.91	0.73	2.17	6.97	4.83	6.10	0.81	16	45.63
	10.15	No.4	10.65	2.84	0.60	1.77	3.66	2.54	7.30	0.97	11	54.55
	13.15	No.5	9.96	2.66	0.65	1.91	5.76	3.99	4.61	0.62	13	50.00
	**13.53**	**Average**	**10.65**	**2.84**	**0.67**	**1.98**	**4.80**	**3.33**	**5.38**	**0.72**	**13**	**51.54**
S2.0	0.000001	No.1	4.38	-	0.25	-	1.21	-	7.69	-	26	9.62
	0.000001	No.2	4.62	-	0.20	-	1.08	-	6.28	-	22	9.09
	0.000001	No.3	4.00	-	0.23	-	1.00	-	7.39	-	24	9.58
	**0.000001**	**Average**	**4.33**	**-**	**0.23**	**-**	**1.10**	**-**	**7.12**	**-**	**24**	**9.58**
	15.40	No.1	11.68	2.70	0.32	1.42	3.15	2.87	6.68	0.94	25	12.80
	17.86	No.2	12.13	2.80	0.25	1.11	1.54	1.40	9.50	1.33	22	11.36
	13.51	No.3	11.56	2.67	0.35	1.53	2.76	2.52	6.17	0.87	24	14.58
	11.52	No.4	12.86	2.97	0.37	1.63	2.06	1.88	5.77	0.81	35	10.57
	16.32	No.5	12.04	2.78	0.33	1.43	2.02	1.84	7.78	1.09	31	10.65
	**14.92**	**Average**	**12.06**	**2.78**	**0.32**	**1.42**	**2.31**	**2.10**	**7.18**	**1.01**	**27.4**	**11.68**

**Table 6:** Tensile properties of HP-HFRCC.

ID.	Strain Rate	Specimen No.	$\mathrm{Tensile}\;\mathrm{Strength}\;(\sigma_{ts})$		$\mathrm{Strain}\;\mathrm{Capacity}\;(\delta_{ts})$		$\mathrm{Peak}\;\mathrm{Toughness}\;(T_{p})$		$\mathrm{Softening}\;\mathrm{Toughness}\;(T_{s})$		Number of Cracks	Average Crack Width
	/s		MPa	DIF	%	DIF	N·m	DIF	N·m	DIF	EA	µm
H1.5 S0.5	0.000001	No.1	3.97	-	0.39	-	1.78	-	9.01	-	13	30.00
	0.000001	No.2	4.20	-	0.40	-	1.99	-	9.51	-	14	28.57
	0.000001	No.3	3.61	-	0.37	-	1.55	-	8.93	-	12	30.83
	**0.000001**	**Average**	**3.93**	**-**	**0.39**	**-**	**1.77**	**-**	**9.15**	**-**	**13**	**30.00**
	11.40	No.1	10.71	2.73	0.82	2.10	7.91	4.47	6.46	0.71	19	43.16
	17.17	No.2	11.34	2.89	0.58	1.50	6.59	3.72	7.74	0.85	18	32.22
	14.94	No.3	11.94	3.04	0.51	1.32	4.36	2.46	11.97	1.31	17	30.00
	11.00	No.4	11.48	2.92	0.50	1.29	4.58	2.59	13.29	1.45	17	29.41
	13.83	No.5	11.08	2.82	0.52	1.33	4.22	2.38	9.24	1.01	19	27.37
	**13.67**	**Average**	**11.31**	**2.88**	**0.59**	**1.51**	**5.53**	**3.12**	**9.74**	**1.06**	**18**	**32.78**
H1.0 S1.0	0.000001	No.1	3.77	-	0.31	-	1.42	-	9.65	-	19	16.32
	0.000001	No.2	4.48	-	0.25	-	1.32	-	10.87	-	22	11.36
	0.000001	No.3	4.19	-	0.33	-	1.63	-	9.58	-	23	14.35
	**0.000001**	**Average**	**4.15**	**-**	**0.30**	**-**	**1.46**	**-**	**10.03**	**-**	**21.3**	**14.09**
	20.59	No.1	12.23	2.95	0.81	2.76	6.50	4.46	10.47	1.04	25	32.40
	14.29	No.2	11.90	2.87	0.77	2.59	6.35	4.36	12.00	1.20	23	33.48
	12.02	No.3	11.38	2.74	0.94	3.18	9.22	6.33	12.34	1.23	27	34.82
	10.73	No.4	12.49	3.01	0.88	3.00	9.34	6.41	10.80	1.08	26	33.85
	15.25	No.5	12.10	2.92	0.72	2.45	7.16	4.92	14.85	1.48	25	28.80
	**14.58**	**Average**	**12.02**	**2.90**	**0.82**	**2.80**	**7.71**	**5.30**	**12.09**	**1.21**	**25.2**	**32.54**
H0.5 S1.5	0.000001	No.1	4.51	-	0.23	-	1.25	-	9.31	-	28	8.21
	0.000001	No.2	4.33	-	0.20	-	1.03	-	8.64	-	26	7.69
	0.000001	No.3	4.69	-	0.19	-	1.07	-	8.12	-	24	7.92
	**0.000001**	**Average**	**4.51**	**-**	**0.21**	**-**	**1.12**	**-**	**8.69**	**-**	**26**	**8.08**
	21.10	No.1	13.41	2.97	0.28	1.34	2.00	1.79	15.38	1.77	28	10.00
	10.26	No.2	12.62	2.80	0.29	1.38	2.67	2.39	11.52	1.33	33	8.79
	12.83	No.3	11.85	2.63	0.33	1.60	2.32	2.08	14.25	1.64	29	11.34
	10.64	No.4	12.27	2.72	0.27	1.31	2.03	1.82	12.16	1.40	27	10.00
	12.18	No.5	12.85	2.85	0.23	1.11	1.99	1.78	17.42	2.00	27	8.52
	**13.40**	**Average**	**12.60**	**2.79**	**0.28**	**1.35**	**2.20**	**1.97**	**14.15**	**1.63**	**28.8**	**9.72**
